# Epidemiology of posterior urethral injury among adults with traumatic pelvic ring disruptions: a 10-year retrospective review from a trauma care centre in Southeast Nigeria

**DOI:** 10.11604/pamj.2023.45.43.34603

**Published:** 2023-05-17

**Authors:** Ikenna Ifeanyi Nnabugwu, Oke Righteous Obadaseraye, Solomon Kenechukwu Anyimba, Chinwe Andrea Nnabugwu, Obinna Nnabuife Anikwe

**Affiliations:** 1 Urology Unit, Department of Surgery, University of Nigeria Teaching Hospital, Ituku-Ozalla, Enugu, Nigeria,; 2 Department of Orthopaedics and Trauma, National Orthopaedic Hospital Enugu, Enugu, Nigeria

**Keywords:** Epidemiology, polytrauma, pelvic fracture, pelvic fracture urethral injury, trauma centre

## Abstract

**Introduction:**

posterior urethral injuries can occur in polytrauma settings, and may contribute to morbidity post-trauma. The aim of this study is to determine the occurrence of pelvic fracture urethral injury (PFUI) in adult polytrauma patients who were successfully stabilized and to appraise the nature of associated injuries.

**Methods:**

the medical records of stabilized polytrauma patients≥ 18 years of age from January 2010 to December 2019 were retrospectively reviewed focusing on those presenting with bony pelvis disruptions. Injuries were categorized using the injury severity scale (ISS) while bony pelvis disruptions were classed according to the Young-Burgess classification. Data on the demography of the patient, mechanism of injury, nature, and severity of injuries, class of pelvic fracture-disruption, and urethral integrity were collected and analyzed accordingly.

**Results:**

of 111 patients with bony pelvis disruptions, 95 of them had adequate information and were included in our analysis. The mean age of participants was 37.3 ± 11.8 years and most of them were males (87.4%). Blunt pelvic trauma occurred in 96.8%. Lateral compression pelvic injuries were prevalent at 39.0%. In 54.7% of the patients, the injury severity score (ISS) was ≥ 27. At 25.3% and 24.2% respectively, the abdomen and the lower extremities most frequently sustained a grade ≥ 3 injuries (abbreviated injury scale (AIS) ≥3). At a rate of 2.1%, spinal cord injury was the least observed. In the 10 years, there were 6 PFUI among 83 stabilized polytraumatized men with mean ISS of 35.5 ± 8.3. The incidence rate of PFUI was 0.6 per 8.3 pelvic disruptions in men per year. Symphysis pubis disruption or fracture of the pubis or both was consistently seen in all PFUI. Higher ISS significantly relates to PFUI (p <0.001). The mechanism of bony pelvis disruption and the class of bony pelvis injury are determined by the severity and trajectory of the impact apparently relates to PFUI only through fracture-disruption of the pubic symphysis or the pubis.

**Conclusion:**

about 7.2% of men presenting with traumatic disruption of the bony pelvis in polytrauma setting sustain PFUI. In polytrauma settings, PFUI should be suspected in cases of fracture-disruption of the pubis or symphysis pubis from any mechanism.

## Introduction

Disruption of the bony pelvis constitutes about 3% of all skeletal injuries in major trauma [1]. It usually occurs in the context of high-energy impact resulting in multiple injuries of varying severity within and outside the bony pelvis [2]. The urethra is recognized as one of the organs that could be injured in association with pelvic injury due to its proximity and attachment to the pelvic ring. Urethral injury from disruptions of the bony pelvis known as pelvic fracture urethral injury (PFUI) typically involves the posterior urethra and is globally reported more commonly in younger men [3,4]. Reportedly, disruptions of the bony pelvis are observed in 7-20% of high-energy polytrauma emergency presentations to the hospital [5]. Similarly, 5 - 10% of all presentations with major pelvic fracture have pelvic fracture urethral injury (PFUI) constituting about 0.6% of all hospital presentations with major trauma [3,6]. Concomitant injuries in traumatic disruptions of the bony pelvis increase the risk of mortality in the immediate period, and of morbidity in the long term [2]. When recognized promptly and managed appropriately, morbidity is decreased [6,7]. So, as part of the evaluation for associated injuries in the care of patients presenting with major pelvic trauma, early recognition of concomitant posterior urethral injury helps avert further damage to the urethra thereby minimizing the attendant morbidity [6,8].

Controlling for severity and mechanism of injury as well as the age of the individual, Basta *et al*. observed that radiographic finding of displaced fracture of the inferomedial pubis or of pubic symphysis diastasis independently predicted concomitant pelvic fracture urethral injury [9]. Similarly, the work by Johnsen *et al*. shows that though only 1.5% of patients with fractures involving the pubic arch sustained the concomitant urethral injury, about 87% of the observed PFUI occurred in those that actually sustained a fracture of the pubic arch [10].

Blood at the urethral meatus or inability to void despite the urge to do so, draw attention to the possibility of concomitant urethral injury [11,12]. When identified early, these features guide the trauma care physician in instituting appropriate measures. However, these features may not be promptly manifest, hence recognizable radiographic features from plain radiographs or computed tomography (CT)-scan of the pelvis help guard against further injury to the urethra inadvertently [9,10].

This study aims to determine the rate of and the fracture patterns in pelvic fracture urethral injury (PFUI) in adults presenting to a tertiary trauma care centre in Southeast Nigeria.

## Methods

**Study design:** this was a retrospective review of the medical records of all clinically-stabilized polytraumatized adult patients who sustained radiographically obvious disruption of the bony pelvis from January 2010 to December 2019.

**Setting:** the study was undertaken at the University of Nigeria Teaching Hospital, a tertiary trauma care centre in Southeast Nigeria.

**Participants:** adult patients who were clinically stable on presentation, or after initial resuscitation to have their bony pelvis evaluated radiologically. These patients must have been evaluated with a plain radiograph of the pelvis, or CT-scan of the pelvis, or both for the suspected bony pelvis disruption as part of protocol of care for polytraumatized patients. Where urethral injury was suspected from the primary survey due to urethral bleeding, inability to void urine despite a full urinary bladder, or leakage of urine into the perineum, suprapubic cystostomy was indicated after failed single attempt at urethral catheterization. Urethrography (retrograde and voiding) was subsequently done electively as an interval investigation to assess urethral integrity.

**Exclusion criteria:** patients below 18 years and those who couldn´t make it through primary survey and initial resuscitation were excluded.

**Variables:** the age and sex of the patients as well as the class of the bony pelvis disruption according to Young-Burgess classification were noted [13]. The severity of concomitant injuries captured using the abbreviated injury scale (AIS) was also taken note of, and from this, each patient´s injury severity score (ISS) was calculated [14]. The presence or not of concomitant PFUI was documented.

**Data sources:** the medical records of the accident and emergency unit of the tertiary institution. Available information needed for the analyses was curled up, between May and July 2021, from the records of individual patients that met the inclusion criteria.

**Sample size:** this retrospective study included the medical records of all patients who had traumatic disruption of the bony pelvis in a polytrauma setting for a period of 10 years in a single institution.

**Statistical analysis:** descriptive statistics were used to define the mean age and the rate of PFUI as well as the rates and severity of concomitant injuries to other regions of the body. Crosstab analyses (for chi-square) were deployed to study the association between traumatic disruption of the bony pelvis and concomitant injuries including pelvic fracture urethral injury. Values of p lower than 0.05 were taken to indicate significant observations. All analyses were done using IBM SPSS Statistics ver. 21.0 (IBM Co., Armonk, NY, USA).

**Ethical approval:** this was obtained from the University of Nigeria Teaching Hospital Bioethics Committee (NHREC/05/01/2008B-FWA00002458-1RB00002323) granted participant´s consent waiver, and approved of the study.

## Results

During the period under review, 111 patients were identified to have radiographic evidence of disruption of the bony pelvis among successfully stabilized polytraumatized patients. However, 95 patients (85.6%) between the ages of 18 years and 89 years (mean 37.3 ± 11.8 years), had adequate information to be included for further analysis. There were 83 (87.4%) males and 12 (12.6%) females.

Blunt pelvic injury from high energy impact was prevalent: 80 patients (84.2%) were involved in an auto vehicular crash, 8 patients (8.4%) fell from a height and 3 patients (3.2%) were fallen upon by heavy objects. Fifty-two patients (54.7%) had an injury severity score (ISS) of at least 27. At the rates of 25.3% (24 of 95) and 24.2% (23 of 95) respectively, the abdomen and the lower extremities most frequently sustained at least a grade 3 abbreviated injury scale (AIS ≥3) injury in association with a bony pelvis disruption. On the contrary, at a frequency of 2.1% (2 of 95), spinal injury with cord involvement was the least frequently observed concomitant injury.

There were 6 PFUI over the 10-year review period giving a gross hospital-based incidence rate of 0.6 PFUI per 8.3 pelvic disruptions in men per year. Put differently, 7.2% of men presenting with traumatic disruption of the bony pelvis in polytrauma setting sustained PFUI. Each had a suprapubic cystostomy for urinary diversion. Regarding the class of bony pelvis disruption sustained. Table 1 shows the frequency distribution of the Young-Burgess classes of observed fracture patterns.

**Table 1:** gender distribution of the observed bony pelvis disruptions

Young-Burgess class of observed bony pelvis disruptions	Female n (%)	Male n (%)	Total n (%)
**Anterior-posterior compression (APC)**			
APC I	2 (2.1%)	5 (5.3%)	7 (7.4%)
APC II	2 (2.1%)	11 (11.6%)	13 (13.7%)
APC III	0	1 (1.1%)	1 (1.1%)
**Lateral compression injuries (LC)**			
LC I	2 (2.1%)	8 (8.4%)	10 (10.5%)
LC II	3 (3.2%)	19 (20.0%)	22 (23.2%)
LC III	1 (1.1%)	4 (4.2%)	5 (5.3%)
**Other fracture disruptions of the pelvis**			
Isolated ramus fracture (IRF)	2 (2.1%)	21 (22.1%)	23 (24.2%)
Isolated acetabular fracture (IAF)	0	7 (7.4%)	7 (7.4%)
Isolated ilial fracture (IIF)	0	3 (3.2%)	3 (3.2%)
Unspecified	0	4 (4.2%)	4 (4.2%)
Total	12 (12.6%)	83 (87.4%)	95 (100%)

APC: anterior-posterior compression; LC: lateral compression; IRF: isolated ramus fracture; IAF: isolated acetabular fracture; IIF: isolated ilial fracture. Males are more frequently affected, and lateral compression (LC) injuries especially the type II (LC II) subtype are observed more commonly in blunt traumatic injuries of the bony pelvis

In crosstab analyses, PFUI was more likely to be seen among patients with more severe injuries (χ^2^ 40.4; df 3; p <0.001). There was no evidence that the nature of the trauma leading to the bony pelvis disruption (χ^2^0.99; df 4; p 0.91) and the class of bony pelvis disruption according to Young-Burgess classification (χ^2^10.78; df 9; p 0.29) predicted the occurrence of PFUI among these polytraumatized men.

Focusing on the 6 men that sustained PFUI (Table 2), the mean age of these patients was 37.8 ± 8.3 years and the mean of the estimated injury severity score (ISS) was 35.5 ± 8.3, motor vehicular crash was the cause in 83.3% while anterior-posterior compression (APC) injuries of the bony pelvis were identified in 66.7%. There was radiographic evidence of symphysis pubis disruption of various dimensions in each of the 6 men.

**Table 2:** clinical characteristics of the patients presenting with PFUI

Patient identity	Age (years)	Cause of injury	Young-Burgess (Y-B) class	Injury severity scale (ISS)
1.	28	MVC	APC I	36
2.	45	FFH	LC II	48
3.	32	MVC	APC II	25
4.	38	MVC	APC II	41
5.	34	MVC	LC II	34
6.	50	MVC	APC II	29
Mean	37.8 ± 8.3			35.5 ± 8.3

APC: anterior-posterior compression; LC: lateral compression; MVC: motor vehicular crash; FFH: fall from height; PFUI: pelvic fracture urethral injury

## Discussion

Traumatic disruption of the bony pelvis is known to occur as a consequence of high-energy impact on the body which usually results in concomitant injuries of varying severity to other regions of the body [2]. In this setting as in many other settings, especially those with a relatively young population, motor vehicular crash is observed to be the most prevalent agent of this high-energy impact, and by implication, the most frequent cause of traumatic disruption of the bony pelvis [15,16]. There is some evidence from this review, in line with existing knowledge, that the male posterior urethra is more at risk, compared to the female urethra, of sustaining an injury in the event of bony pelvis disruption from major trauma [11]. Among polytraumatized men that were successfully stabilized in this tertiary care centre, the observed annual rate of PFUI is 0.6 per 8.3 bony pelvis disruptions. Specifically, this is approximately 1 PFUI per 14 bony pelvis disruptions in such men. This rate is similar to reports from Bhatt *et al*. [17] in 2018 and Horiguchi *et al*. in 2019 [11].

As a group, the lateral compression (LC) fracture disruptions of the bony pelvis are slightly more prevalent than the anterior-posterior compression (APC) fracture disruptions or the various isolated fracture disruptions (Table 1). This is similar to the report by Hossain *et al*. [16]. However, this higher prevalence of LC injuries is not observed among the patients who sustained PFUI (Table 2). On the other hand, all 6 patients presenting with PFUI reveal symphysis pubis disruption or pubic ramus fracture, or both. In many studies on PFUI, the association between disruption of the symphysis pubis and concomitant posterior urethral injury is reported irrespective of pelvic fracture patterns or classification system adopted [8,12,18].

Most of the patients in this review (54.7%) presented with major injuries in some other regions of the body, an observation that does not vary from findings elsewhere [15,19]. The abdomen and the lower limbs are the regions commonly injured in concomitance with traumatic injuries to the bony pelvis. This is similar to observations from other reviews such as from the Netherlands [15] and from Germany [19], and could be explained by the proximity of these regions of the body to the region of the pelvis.

The occurrence of multiple injuries with a mean ISS of 35.5 ± 8.3 (Table 2) among these patients presenting with PFUI corroborates the high-energy impact. Fixed to the pubic bones at the symphysis pubis through the puboprostatic ligament, the relatively immobile, poorly distensible, membranous urethra is at risk of avulsion to various degrees from the more mobile bulbous urethral segment at the bulb-membranous junction by the impact that results in traumatic disruption of the anterior pelvic ring [20-22].

Granted that the cases of PFUI from this single-centre review are few, thereby mitigating against the generalization of findings thereof, the 10-year span of the review seems adequate. In similarity to the observations of Basta *et al*. [9] and Johnsen *et al*. [10], PFUI is more likely to be seen among polytraumatized men presenting with fracture disruption of the anterior pelvic ring. Therefore, the observation of anterior pelvic ring disruption in a polytraumatized man is an indication that high energy impact coursed through this part of the bony pelvis and that there could be PFUI [20,23].

It is understood that some of these men may have symptoms or signs suggesting urethral injury such as brisk urethral bleeding, distorted urine flow, or outright inability to void which accordingly will increase the suspicion of concomitant urethral injury [11,12]. However, in the absence of these symptoms, the observation from a pelvic radiograph of symphysis pubis disruption or pubic ramus fracture in a polytraumatized man should make the clinician suspect PFUI and plan further evaluations and interventions along that line.

**Limitations:** this is a retrospective review with its attendant limitations. It has not taken into account the details of the injuries in the missing medical records as well as among the patients that could not be resuscitated and stabilized for further evaluation. Findings from this single-centre experience with a limited number of PFUI may only be generalized with caution.

## Conclusion

Major traumatic disruptions of the bony pelvis occur in the context of polytrauma, and concomitant abdominal and lower extremity injuries are prevalent. One of every 14 men who sustained polytrauma, including bony pelvis disruption, has a concomitant posterior urethral injury. This is particularly so where concomitant injuries are assessed to be severe, and where the disruption of the bony pelvis is diastasis of the symphysis pubis, fracture of the pubic ramus, or both. A high index of suspicion for possible posterior urethral injury in these clinical scenarios is invaluable.

### What is known about this topic

- *Posterior urethra can be injured in the setting of polytrauma;*
- *Posterior urethral injury tends to occur with traumatic disruption of the bony pelvis;*
- *Pelvic fracture urethral injury (PFUI) is very rare in females*.

### What this study adds

- *Pelvic fracture urethral injury (PFUI) can be observed in both anteroposterior compression (APC) and lateral compression (LC) injuries;*
- *Disruption of the symphysis pubis is a consistent pointer to the presence of PFUI in men;*
- *The severity and trajectory of the impact leading to the class of injury predict the occurrence of PFUI*.
